# The role of TGF-β or BMPR2 signaling pathway-related miRNA in pulmonary arterial hypertension and systemic sclerosis

**DOI:** 10.1186/s13075-021-02678-6

**Published:** 2021-11-25

**Authors:** Bei Xu, Guanhua Xu, Ye Yu, Jin Lin

**Affiliations:** Department of Rheumatology, The First Affiliated Hospital, Zhejiang University School of Medicine, #79 Qingchun Road, Hangzhou, Zhejiang Province People’s Republic of China 310003

**Keywords:** Pulmonary arterial hypertension, miRNAs, Systemic sclerosis

## Abstract

Pulmonary arterial hypertension (PAH) is a severe complication of connective tissue disease (CTD), causing death in systemic sclerosis (SSc). The past decade has yielded many scientific insights into microRNA (miRNAs) in PAH and SSc. This growth of knowledge has well-illustrated the complexity of microRNA (miRNA)-based regulation of gene expression in PAH. However, few miRNA-related SSc-PAH were elucidated. This review firstly discusses the role of transforming growth factor-beta (TGF-β) signaling and bone morphogenetic protein receptor type II (BMPR2) in PAH and SSc. Secondly, the miRNAs relating to TGF-β and BMPR2 signaling pathways in PAH and SSc or merely PAH were subsequently summarized. Finally, future studies might develop early diagnostic biomarkers and target-oriented therapeutic strategies for SSc-PAH and PAH treatment.

## Introduction

Systemic sclerosis (SSc) is a complex, multisystem disease characterized by fibrosis and excessive collagen deposition within the skin and internal organs, chronic inflammation, autoimmune dysregulation, and microvascular endothelial dysfunction [1]. With the advent of angiotensin-converting enzyme inhibitors to treat the SSc renal crisis, SSc-associated pulmonary arterial hypertension (SSc-PAH) has emerged as a leading cause of morbidity and mortality of premature deaths. Therefore, SSc-PAH has a poor prognosis [2–9]. PAH is the leading cause of death in SSc and affects up to 12% of all patients with SSc, with a 50% mortality rate within 3 years of PAH diagnosis [10]. Annual mortality of PAH remains high at up to 10% in idiopathic PAH [11–14]. PAH is defined by an elevated mean pulmonary artery pressure (mPAP) of > 25 mmHg, with a pulmonary capillary wedge pressure of < 15 mmHg. The prevalence of SSc-PAH among patients with SSc varies but is between 10 and 12% [10].

SSc-PAH occurs as a consequence of progressive remodeling of the small- to medium-sized pulmonary vasculature. The exact mechanisms of disease progression are still unclear, but it is believed that inflammation and endothelial injury are common precursors [15, 16]. In addition, pulmonary artery vasoconstriction and cellular proliferation occur during PAH progression. Further ischemia-reperfusion injury in the pulmonary vasculature promotes additional cytokine release, furthering vascular remodeling, fibrosis, and intraluminal microthrombosis. The end outcome is a progressive increase in pulmonary vascular resistance, pulmonary arterial pressure, and right ventricular (RV) pressure overload [17].

Generally, the PAH is thought to be characterized by a disbalance in transforming growth factor-beta (TGF-β) and bone morphogenetic protein (BMP) signaling [18]. Bone morphogenetic protein receptor type II (BMPR2) is known to be involved in osteogenesis and cell differentiation. The BMPR2 pathway inhibits SMC cell proliferation within the pulmonary circulation, primarily within the small pulmonary arterioles. When mutated, BMPR2 is associated with an increased susceptibility to develop PAH [19]. TGF-β signaling pathways have a complex and opposing effect on tissues [18]. TGF-β superfamily ligands modulate a wide range of developmental programs, cellular processes, and disease states. TGF-β1 has vital physiological roles in embryonic development, angiogenesis, wound healing, inflammation, and immune cell function by T cell regulation and differentiation. However, excessive TGF-β1 production is associated with fibrotic lung diseases [20]. Dysregulated TGF-β signaling potentially causes inflammation, autoimmune disorders, fibrosis, cancer, or PAH [21].

The mortality and therapeutic response in SSc-PAH are worse than idiopathic pulmonary arterial hypertension (IPAH) and might partially be due to its multifaceted underlying mechanisms and the multisystem nature [16]. Therefore, a multidisciplinary approach composing an earlier diagnosis or therapy with biomarkers and better characterization of the clinical phenotypes of SSc-PAH could be helpful in SSc-PAH management. In addition, more understanding of the potential biomarkers involved with PAH and SSc, such as microRNA (miRNAs), might benefit for predicting the presence and progression and identify the therapeutic target of PAH and SSc-PAH.

miRNAs represent 21 to 25 nucleotide non-coding small RNAs that negatively regulate gene expression at the post transcription level. Thus, miRNAs are regulatory molecules addressed as potential biomarkers and therapeutic targets in rheumatic diseases. However, previous studies found and discussed few miRNAs related to transforming growth factor-beta (TGF-ß) and Bone Morphogenetic Protein Receptor Type II (BMPR2) signaling in SSC-PAH. This study reviews the miRNAs relating to these two signaling in PAH and/or SSc from previous literature and further investigates their pathological and regulatory roles in SSc-PAH.

### Narrative review process

**Fig. 1:**
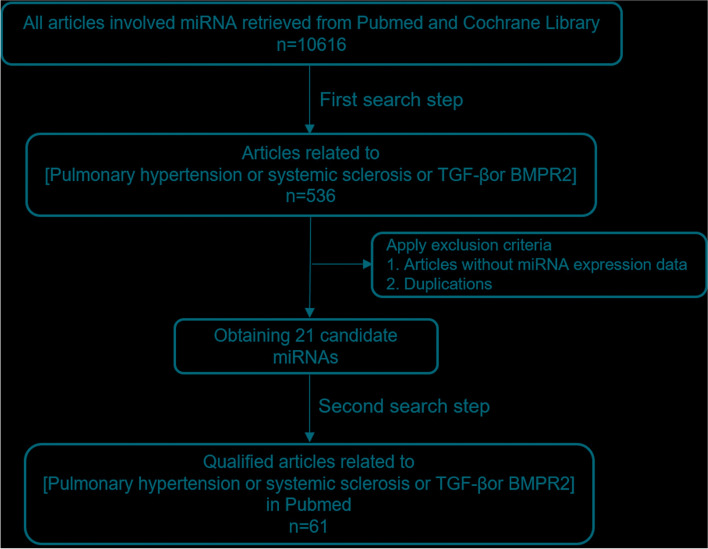
Flowchart of searching candidate miRNAs and related articles included in this study

Twenty-one candidate miRNAs were obtained firstly by the first search in PubMed (https://pubmed.ncbi.nlm.nih.gov/) and Cochrane Library (https://www.cochranelibrary.com/) using the keywords of miRNA, PAH, SSc, TGF-ß, and BMPR2 (Fig. 1). The 31 literature related to these miRNAs were obtained by the second search in PubMed and listed in Table 1.

**Table 1:** A List of microRNAs in this study

miRNA	Experimental system	Targets	Function	References
Putative miRNAs related to PAH and SSc				
miR-21	Human PAECs and PASMCs	KLF4, Smad-8	PASMC differentiation and inhibition of PASMC proliferation	[22]
miR-21	Human PAECs Rodent model of PH	BMPR2 PDCD4/caspase-3 Proliferating cell nuclear antigen cyclin D1 Bcl-xL DDAH1	PAH	[23–26]
miR-21	Fibroblasts	SMAD7	Skin fibrosis	[27]
let-7g	Human patients with SSc-PH	multiple	PAH	[28]
let-7a	Human or mouse skin fibroblasts	Type I collagen	Tissue fibrosis in the skin	[29]
miR-155	Skin of mice	CK1α SHIP-1	Dermal fibrosis	[30]
miR-29a	Skin biopsy and fibroblast samples from SSc patients and healthy controls The mouse model of bleomycin-induced skin fibrosis	Type I and type III collagen	Fibrogenesis of SSc	[31]
miR-140-5p	Marrow stromal cells	TGF-βR1	TGF-βR1 regulation Adipocyte differentiation	[32]
miRNA related to TGF-β-signaling pathway				
miR-155	Lung fibroblasts and PBMC from SSc-ILD patients	Multiple	Dysregulated lung gene expression	[30]
miR-145	Normal and cutaneous scleroderma skin tissues Human fibroblasts	SMAD7 SMAD3 COL1A1	SSc	[33]
miR-145	PAH patients Human PASMCs Hypoxia-induced PAH rats	ABCA1	PASMCs proliferation and migration	[32]
miR-221-3p	Human PASMC PAH animal models	AXIN2	PASMC proliferation	[34]
miR-124	Pulmonary vascular and circulating progenitor endothelial cells isolated from patients	PTBP1	Metabolic and proliferative abnormalities in PAH ECs	[35]
miR-124	Human PASMCs Nonfamilial human PAH PAH mouse and rat models	NFATc1 CAMTA1 PTBP1 STAT3/NFAT signaling	PASMC proliferation, prodifferentiation, and survival Inflammation Pulmonary vascular remodeling	[36]
miR-143	PAH patients Human PASMCs Hypoxia-induced PAH rats	ABCA1	PASMC proliferation and migration	[32]
miR-143-3p	Calf models of PAH PAH patients Mice	Multiple	PH	[37]
miR-181a-5p	Human pulmonary artery endothelial cells PAH mice	Multiple	Vascular remodeling	[38]
miR-29 miR-29b	Mice Mice pulmonary fibroblasts	TGF-β CTGF SMAD3	Pulmonary fibrosis	[39]
miR-455-3p-1	Normal and PAH patients	FGF7	Inhibit the proliferation of PASMCs	[40]
miR-223-3p	PASMCs Rat	ITGB3	Pulmonary vascular remodeling	[41]
miR-17/92 family	HEK293 Rodent model of PH	BMPR2 STAT3	Development of PH	[42]
miRNA related to BMPR2 signaling pathway				
miR-204	PASMCs in both human and rodent PAH	SHP2 NFAT	PAH-PASMC proliferation and resistance to apoptosis	[43]
miR-130/301 family	Pulmonary vessels and plasma from mammalian models and PH patients PAECs and PASMCs in mice model	PPARγ	Cell proliferation in PH	[44]
miR-130/301 family	Human PASMCs and PAECs Mice blood and lung tissue	PPARγ	Cell proliferation in PH	[45]
miR-130/301 family	Human PAECs	PPARγ LRP8	Pulmonary vascular stiffening Extracellular matrix remodeling	[46]
miR-424 (322)	The blood of PAH patients Hypoxia-induced PAECs Monocrotaline rat model of PH	SMURF1	Afterload of the right ventricle	[47]
miR-17/92 family	Human PASMCs	PDLIM5	PASMC proliferation and differentiation	[48]
miR-20a	Mice Human PASMCs	BMPR2	Prevent pulmonary arterial vascular remodeling	[49]

*PAH* pulmonary arterial hypertension, *SSC* systemic sclerosis, *PAEC* pulmonary artery endothelial cell, *PASMC* pulmonary artery smooth muscle cell, *KLF4* Kruppel Like Factor 4, *Smad-8* Mothers against decapentaplegic homolog 8, *PH* pulmonary hypertension, *BMPR2* Bone Morphogenetic Protein Receptor Type II, *PDCD4* Programmed Cell Death 4, *Bcl-xL* B cell lymphoma-extra large, *DDAH1* dimethylarginine dimethylaminohydrolases 1, *SMAD7* Mothers against decapentaplegic homolog 7, *CK1α* casein kinase 1α, *SHIP-1* Src homology 2-containing inositol phosphatase-1, *TGF-βR1* transforming growth factor-beta receptor type 1, *SSc-ILD* systemic sclerosis associated interstitial lung disease, *SMAD7* Mothers against decapentaplegic homolog 7, *SMAD3* Mothers against decapentaplegic homolog 3, *COL1A1* Collagen Type I Alpha 1 Chain, *ABCA1* ATP-binding cassette transporter A1, *AXIN2* Axis inhibition protein 2, *PTBP1* polypyrimidine tract-binding protein 1, *NFATc1* nuclear factor of activated T cells 1, *CAMTA1* calmodulin-binding transcription activator 1, *STAT3* signal transducer and activator of transcription 3, *NFAT* nuclear factor of activated T cells, *TGF-β* Transforming growth factor-beta, *CTGF* connective tissue growth factor, *FGF7* Fibroblast Growth Factor 7, *ITGB3* Integrin alpha-V/beta-3, *SHP2* Src homology region 2 domain-containing phosphatase-2, *PPARγ* Peroxisome proliferator- activated receptor gamma, *LRP8* lipoprotein receptor-related protein 8, *SMURF1* SMAD-specific E3 ubiquitin protein ligase 1, *PDLIM5* PDZ And LIM Domain 5

### The role of TGF-β signaling in PAH

**Fig. 2:**
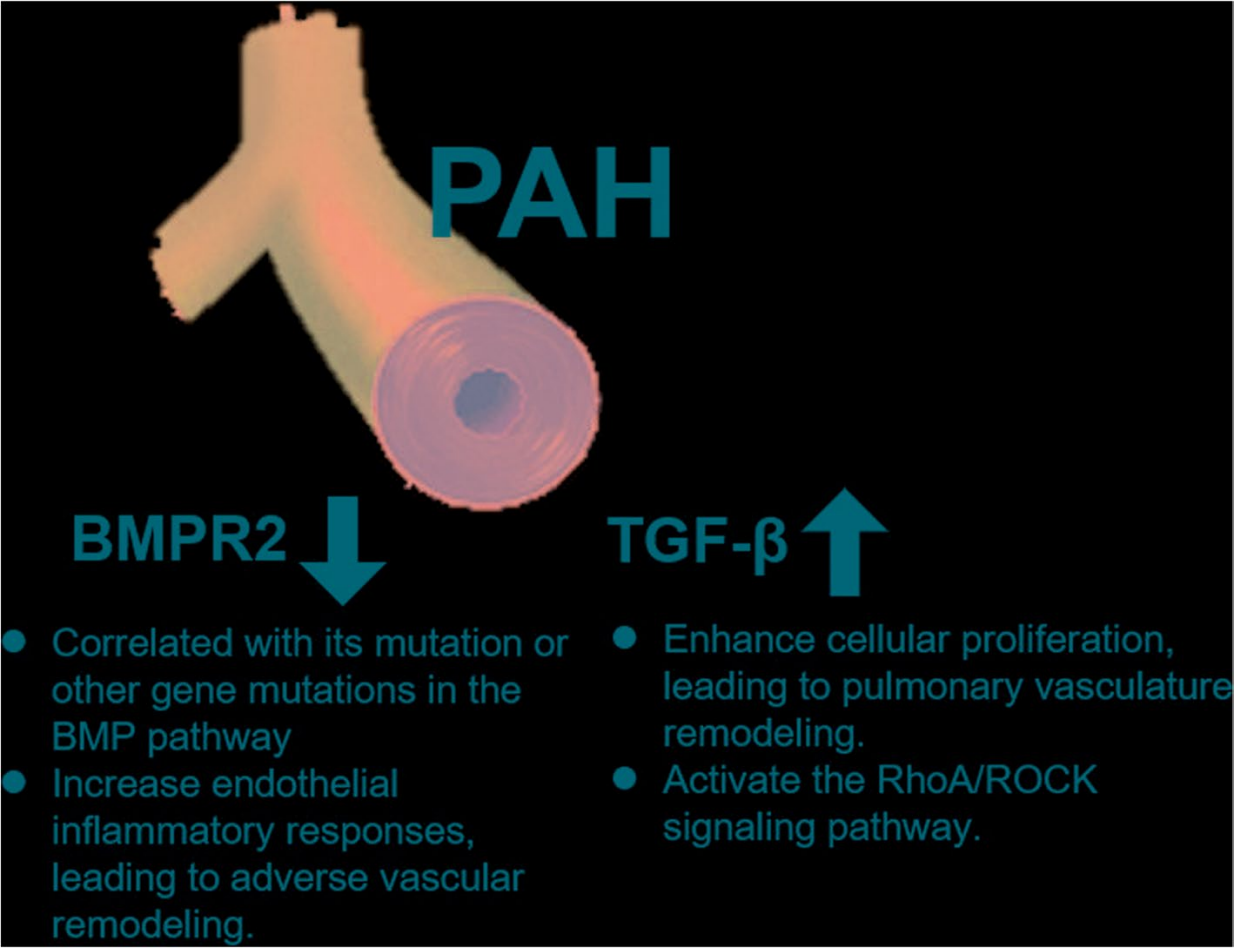
The role of TGF and BMPR2 signaling in PAH. Abbreviation: TGF, transforming growth factor; BMPR2, Bone Morphogenetic Protein Receptor Type II; PAH, pulmonary arterial hypertension

TGF-ß signaling has been strongly implicated in the pathogenesis of PAH. The activation of the TGF-β signal is vital for the formation of PAH [50]. TGF-ß signaling regulates several processes, including cellular proliferation and angiogenesis (Fig. 2). TGF-β is elevated in PAH and implicated in its pathogenesis based on clinical and experimental data [51, 52]. TGF-ß serum concentrations are raised in IPAH patients [18]. The antiproliferative BMP signaling is decreased in the PAH lung, while elevated levels of circulating TGF-β enhance the proliferation of vascular cells leading to occlusive remodeling in the pulmonary vasculature [53]. A study of monocrotaline (MCT)-treated rat model demonstrated that inhibiting the TGF-β pathway with orally active small-molecule transforming growth factor-beta receptor type 1 (TGF-βR1) inhibitor can reduce MCT-induced pulmonary hypertension (MCT-PH) [54]. These findings confirmed that endothelial apoptosis induces pulmonary artery smooth muscle cell (PASMC) growth via TGF-β. Adenovirus-mediated overexpression of TGF-βR1 causes pulmonary fibrosis and PH associated with increased TGF-β signaling in the lung tissue surrounding the remodeled pulmonary blood vessels. TGF-β binding to its receptor activates downstream signaling cascades, such as SMAD proteins. Vascular remodeling in PAH results from smooth muscle cell hypertrophy and proliferation of vascular cells. Increased signaling via TGF-β and its downstream mediators SMAD2/3 has been proposed to drive lung vascular remodeling [55]. A previous study showed that PAH rats injected with TGF-β1 recombinant protein could activate the mRNA and protein expression of RhoA and ROCK, revealing that overexpression of TGF-β1 might activate the RhoA/ROCK signaling pathway and promote the occurrence and development of PAH (Fig. 2) [56].

### The role of BMPR2 in PAH

**Fig. 3:**
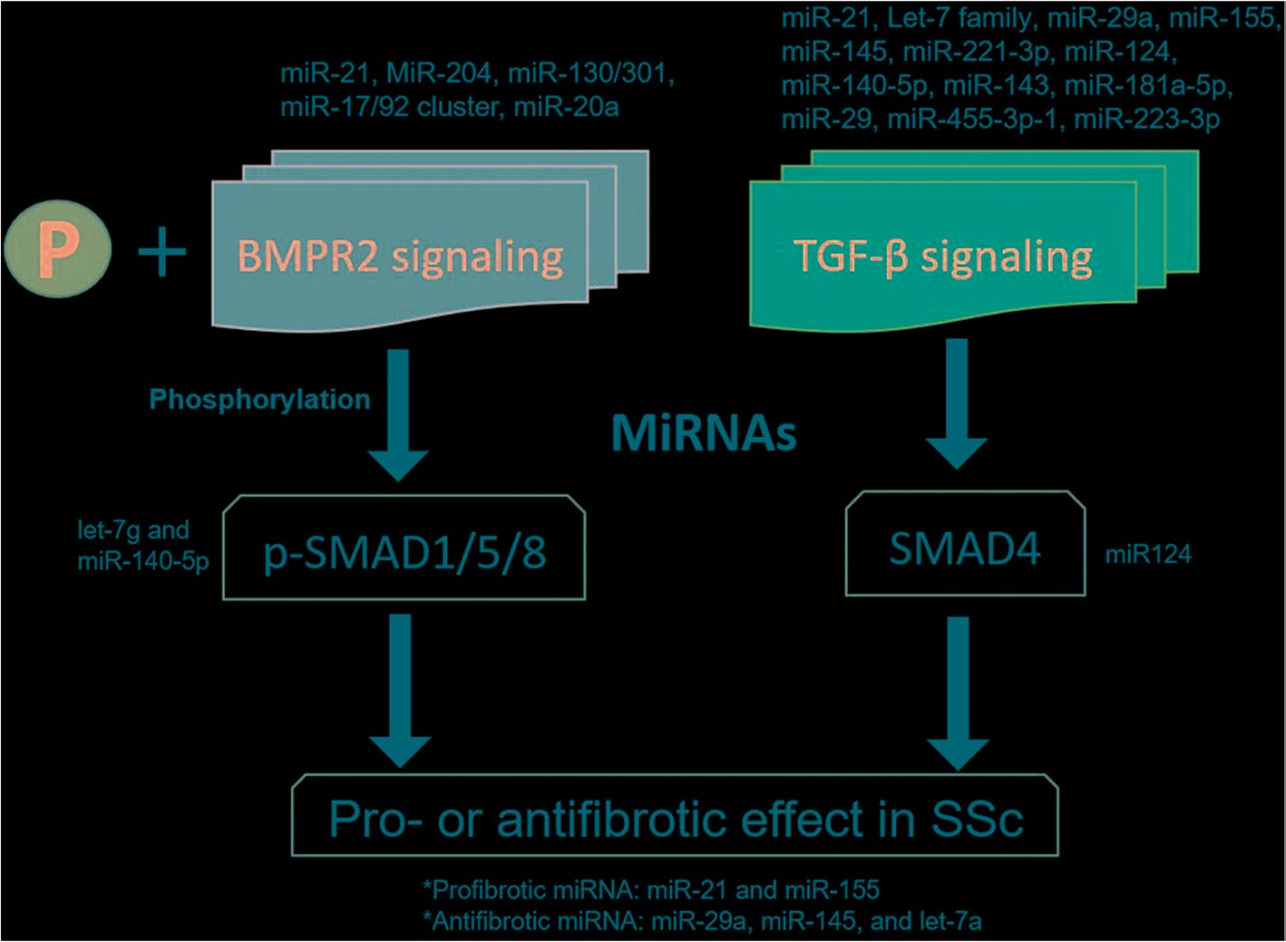
The SSc-related miRNAs in TGF-β signaling and BMPR2 signaling. Abbreviation: TGF-β, Transforming growth factor-beta; BMPR2, SSc, systemic sclerosis; SMAD1/5/8, Mothers against decapentaplegic homolog 1/5/8; SMAD4 Mothers against decapentaplegic homolog 4

BMPR2 is a transmembrane serine/threonine kinase receptor of the BMP pathway, essential for embryogenesis, development, and adult tissue homeostasis. BMP-induced heteromeric complex formation of BMPR2 with BMP type I receptor (BMPR1). BMPR2 subsequently activates BMPR1 by phosphorylation. After that, the activated BMPR1 propagates the signal into the cell through phosphorylation of the SMAD1/5/8 transcription factors (Fig. 3) [57].

A previous study revealed reduced BMPR2 protein in patients with SSc-PAH, and an increased proteasomal degradation of BMPR2 was found in a relevant mouse model [58]. Collectively, these results suggest that TGF-β might impair the BMP signaling through the degradation of its receptor and promote the PAH susceptibility in SSc, which might provide a unifying mechanism across different forms of PAH [58]. Although the BMPR2 pathway is essential for vascular homeostasis and there is a strong correlation between BMPR2 mutations and PAH, the incomplete penetrance of BMPR2 mutations (20–30%) suggests other genetic and environmental factors might contribute to this disease [59]. One BMPR2 splice variant lacks exon 12, which is the largest exon of the gene and encodes the cytoplasmic tail. It has been shown that carriers of this variant are more prone to develop PAH [60]. Furthermore, mutations in other genes in the BMP pathway further strengthen the notion of a causal role for this pathway in PAH (Fig. 2) [61]. Moreover, the co-existence of modifier genes, infections, toxic exposure, inflammation, or alterations in estrogen metabolism has been described [61–64], and some of them were found to downregulate BMPR2 expression. For example, pro-inflammatory cytokines such as tumor necrosis factor α (TNF-α) and interleukin 6 (IL-6) induce miRNA expression that inhibits BMPR2 expression [42]. Furthermore, BMPR2 is essential for maintaining the pulmonary artery endothelial cell lining barrier function, and BMPR2 deficiency increases endothelial inflammatory responses, thereby contributing to adverse vascular remodeling (Fig. 2) [65–67]. Recent study showed BMPR2 expression and downstream signaling is reduced in the lung vasculature of patients with idiopathic and hereditary PAH [68]. Despite increased BMPR2 expression in the lung vasculature, the MCT and SuHx rat models did develop PAH and impaired downstream BMPR2-Smad signaling [68].

### TGF-β and BMPR2 signaling pathway-related miRNA in PAH and SSc

Recently, much attention has been paid to miRNAs as a potential biomarker for PAH [28, 69]. Several clinical factors and biomarkers have been implicated in SSc-associated PAH [48, 70, 71]. In recent reports, miRNAs were proposed as possible novel players in SSc fibrosis, capable of modulating several fibrotic-related genes (Fig. 3) [72]. The aberrant expression of pro-fibrotic and anti-fibrotic miRNAs in SSc might play critical roles in the disease (Fig. 3) [73]. miRNA has initially been thought to function only intracellularly, but recent data suggest that it is also secreted and detected in the circulation [74]. Various miRNAs have been identified to be involved in the pathogenesis of PAH [75]. Some circulating miRNAs were dysregulated in pulmonary hypertensive (PH) and vary according to the severity of PH human [76]. In this review, the miRNAs involved in PAH and SSc were searched in the PubMed and Cochrane Library database, and 21 candidate miRNAs that involved TGF-β and/or BMPR2 signaling pathway were reported (Table 1). These miRNAs were classified by (I) Putative miRNAs related to both PAH and SSc, (II) miRNA related to TGF-b signaling pathway in PAH, and (III) miRNA related to BMPR2 signaling pathway in PAH and described individually for each miRNA.

#### Putative miRNAs related to both PAH and SSc

The miRNAs more evidently related to SSc-PAH are listed in Table 2, classified by apoptosis, cell proliferation, angiogenesis, cell differentiation, cell migration, vasodilation, pulmonary fibrosis, pulmonary hypertension, and pulmonary vascular remodeling. Those miRNAs were found to regulate TGF-β signaling pathway or BMPR2 signaling pathway. Each of these miRNAs is discussed separately by paragraph.

**Table 2:** Pathological mechanisms and putative miRNA in SSc-PAH

Mechanism	Putative miRNA	Potential function	Reference
Apoptosis	miR-21	Reduce the pulmonary endothelial cell apoptosis	[22]
	miR-140-5p	Promote cell apoptosis	[31]
Cell proliferation	miR-21	Antiproliferative effect in PAEC and PASMC	[74]
	miR-140-5p	Inhibit cell proliferation	[31, 77, 78]
Angiogenesis	miR-21	Decrease angiogenesis	[75]
Cell differentiation	miR-140-5p	Promote cell differentiation	[31]
Cell migration	miR-140-5p	Inhibit cell migration	[77, 78]
Vasodilation	miR-21	Increase the pulmonary vasodilation	[75]
Pulmonary fibrosis	miR-21	Target of fibrosis inhibition	[26]
	let-7 family	Reduce fibrosis	[79]
	miR-155	Reduce fibrosis	[32, 80, 81]
Pulmonary hypertension	miR-21	Increase the lung DDAH1and cGMP levels and attenuate pulmonary hypertension	[76]
Pulmonary vascular remodeling	miR-29a	Decrease pulmonary artery pressure and right ventricle hypertrophy index and ameliorate pulmonary vascular remodeling	[29, 82, 83]

Abbreviation: *PAEC* pulmonary artery endothelial cell, *PASMC* pulmonary artery smooth muscle cell, *DDAH1* dimethylarginine dimethylaminohydrolases 1, *cGMP* cyclic guanosine monophosphate

##### miR-21

Several previous studies presented that microRNA-21 (miR-21) might play a central regulatory role for PH and heritable pulmonary arterial hypertension (HPAH) patients [22, 23]. miR-21 could be a critical component of BMP-induced growth suppression of vascular cell proliferation in HPAH 74 and regulate some target genes for attenuating PH such as BMP receptor type 2, programmed cell death 4 (PDCD4)/caspase-3, proliferating cell nuclear antigen, cyclin D1, B cell lymphoma-extra large (Bcl-xL), and dimethylarginine dimethylaminohydrolases 1 (DDAH1) [23–26]. Besides, in terms of TGF-β signaling, miR-21 was revealed to suppress baseline expression of the anti-fibrotic signaling molecule SMAD7, thereby promoting pro-fibrotic activity in spinal fibroblasts [27]. It also exerts a pro-fibrotic effect via Smad7 in SSc dermal fibroblasts [33, 79]. Thus, MiR-21 could be induced by TGF-β and, in turn, downregulates Smad7, which promotes the pro-fibrotic signal of TGF-β. Therefore, miR-21 might be an enhancer that amplifies the effect of TGF-β in SSc fibrosis, and it might be the therapeutic target of SSc-PAH.

##### Let-7 family

Let-7 family miRNAs are thought to be potential biomarkers for the presence and severity of PAH patients with SSc. The expression level of let-7 family microRNAs in skin and lung tissue was lower in SSc patients with PH than those without PH. Let-7b and Let-7d expression levels negatively correlated with the severity of PH in patients with SSc. Let-7e expressed in the lungs of patients with SSc related to TGF-β signal pathway [22, 28, 82]. In the mouse model of bleomycin-induced dermal sclerosis, let-7a expression was downregulated in SSc and localized scleroderma (LSc) skin both in vivo and in vitro, compared with normal or keloid skin. Serum let-7a concentration was significantly decreased in these patients, especially in localized scleroderma patients. Moreover, miRNA injection can improve the skin fibrosis induced by bleomycin in mice. Thus, let-7a-mediated regulation of collagen expression may lead to new therapeutic approaches against SSc and LSc [29].

##### miR-29a

The first report of miRNAs involved in SSc pathogenesis was published by Kawashita et al., showing that miR-29a was detectable in serum and might be reduced in patients with SSc, which was assumed to be relevant an actor for developing SSc in the early disease stage [83]. Although miR-29a targets Collagen Type I Alpha 1 Chain (COL1A1), the significant differences in serum levels of miR-29a between controls and SSc patients were not observed [31]. However, lower miR-29a levels were associated with higher right ventricular systolic pressure in SSc patients, suggesting the involvement of this miRNA in PH’s pathogenesis. Furthermore, a decrease of miR-29a-3p expression was found either in pulmonary adventitial fibroblasts with hypoxia induction or cultured pulmonary adventitial fibroblasts with knockdown hypoxia-inducible factor-1 α or Smad3. Furthermore, miR-29a-3p can significantly decrease pulmonary artery pressure and right ventricle hypertrophy index, ameliorate pulmonary vascular remodeling in hypoxic pulmonary hypertension rats, and suggest regulating pulmonary adventitial fibroblasts hypoxia and preventative and therapeutic potential in hypoxic PH [77]. In mice with overexpression of miR-29a, the TGF-β expression and phosphorylated SMAD2/3 decreased with the downregulation of collagen I and III [84]. These results can suggest that intervention ofmiR-29a may be a therapeutic strategy for attenuating SSc-PAH.

##### miR-155

MircroRNA-155 (miR-155) might play a role in the progression of lung fibrosis in SSc [85]. Increased expression of miR-155 in patients with SSc-systemic sclerosis-associated interstitial lung disease (SSc-ILD) is associated with impaired respiratory function and increased lung fibrosis [85]. miR-155 was found to directly target SMAD2 mRNA by the decreased expressions of SMAD2 [78]. Overexpression of miR-155-5p inhibited the TGF-β1/Smad2/3 signaling pathway, as evidenced by decreased protein expression of TGF-β1, pSmad-2, and pSmad-3 in rat vascular smooth muscle cells [80]. Although miRNA expression is tissue-specific and cell-type-dependent, the circulating fraction of miR-155 may act as a biomarker for SSc [78]. Since miR-155 might act as a regulator of the TGF-β1/Smad2/3 signaling pathway, it may also be a potential therapeutic target for SSc-PAH.

##### miR-145

Downregulation of miR-145 was observed in SSc fibroblasts, while its predicted target Smad3 was upregulated [33]. Thus, MiR-145 may regulate TGF-β signaling through Smad3; however, more mechanistic studies need to confirm this link. A previous study for PAH patients, PASMCs, and hypoxia-induced PAH rats showed that miR-145 could promote hypoxia-induced proliferation and migration of PASMCs by regulating ATP-binding cassette expression subfamily A member 1(ABCA1), which suggested that miR-145 might involve in the pathogenesis of PAH [32]. Another previous report showed that the expression levels of miR-145 and its target proteins such as myocardin, smooth muscle myosin heavy chain were significantly higher in human PAH with concentric lesions than in plexiform ones [81]. Since miR-145 was found to associate with both SSc and PAH, it may also be a potential therapeutic target for SSc-PAH.

#### miRNA related to TGF-β signaling pathway in PAH

##### miR-140-5p

Recent studies have demonstrated a downregulation of microRNA-140-5p (miR-140-5p) levels in treatment-naive patients and experimental models of PAH. In contrast, miR-140-5p could inhibit proliferation and differentiation of HPASMCs and promotes apoptosis in hypoxia, prevent the development of PAH, and attenuated the progression of established PAH [30, 86]. The potential targets of its regulation included DNA methyltransferase 1 (Dnmt1), which can downregulate superoxide dismutase 2 (SOD2) expression, and SMAD-specific E3 ubiquitin protein ligase 1 (SMURF1), which can alter BMP signaling [30, 86]. Besides, the primary target genes of miR-140-5p were mainly located in Notch, TGF-b, PI3K/Akt, and Hippo signaling pathways [87]. TGF-βR1 was found to be one of the direct targets of miR-140-5p. Supplementing miR-140-5p in ST2 bone marrow stromal cells reduced the level of TGF-βR1, while suppression of endogenous miR-140-5p increased TGF-βR1 [88]. In summary, miR-140-5p is an essential regulator in PAH pathology and may serve as a therapeutic target for PAH [34].

##### miR-221-3p

A previous study revealed that elevated expression of MicroRNA-221-3p (miR-221-3p) was observed in lung tissue and PASMC of PAH patients and animal models of PAH and miR-221-3p together with axis inhibition protein 2 (AXIN2) might regulate the proliferation of PASMC [89]. In rat cardiac fibroblasts, inhibition of miRNA-221/222 derepressed TGF-β-mediated pro-fibrotic mothers against decapentaplegic homolog 2 (SMAD2) signaling and downstream gene expression, whereas overexpression of both miRNAs blunted TGF-β-induced pro-fibrotic signaling. The miRNA-221/222 family may target several genes involved in TGF-β signaling, including c-Jun N-terminal kinase 1 (JNK1), TGF-β receptor 1 and TGF-β receptor 2, and ETS proto-oncogene 1 (ETS-1) [90]. Therefore, miRNA-221 might be involved in PAH pathology via TGF-β signaling pathway and tumor suppressor Axin2.

##### miR-124

MicroRNA-124 (miR-124) is considered to regulate the TGF-β signal in human according to previous studies [91]. TGF-β activation can inhibit the expression of miR-124 and promote the expression of downstream Smad4 [35]. The reduced expression of miR-124 was observed in pulmonary vascular and circulating progenitor endothelial cells isolated from PAH patients, leading to the identification of miR-124 as a significant regulator of enhanced endothelial cell glycolysis in PAH via polypyrimidine tract-binding protein (PTBP1) and Pyruvate Kinase M2 (PKM2). Therefore, miR-124 or its targets might be developed for the treatment of PAH [36]. miR-124 could robustly inhibit the nuclear factor of activated T cells (NFAT) reporter activity and decrease both the dephosphorylation and the nuclear translocation of NFAT. It could also inhibit the NFAT-dependent transcription of IL-2 in Jurkat T cells. Furthermore, the overexpression of miR-124 inhibited human PASMC proliferation and maintained its differentiated phenotype by repressing the NFAT pathway. These results imply a potential value for miR-124 in the treatment of PAH [92].

##### miR-143

MicroRNA-143 (miR-143) is considered to regulate the TGF-β signal in human according to previous studies [93]. It controls the proliferation of tracheal smooth muscle cells induced by TGF-β1 through the activation of T cell nuclear factors (Fig. 2) [37]. A previous study showed the protective role of miR-143 in experimental PH in vivo and which could modulate smooth muscle and endothelial cell crosstalk in pulmonary vascular cells, whereas inhibition of miR-143-3p blocked experimental PH. These findings confirm an essential role for the miR-143 in PAH pathobiology [94].

##### miR-181a-5p

The bioinformatics analysis in previous report suggested that miR-181a negatively regulated TGF-βR2. Overexpression of miR-181a and the downregulation of TGF-βR2 promoted the migration and proliferation of gastric cancer cells [95]. miR-181a-5p overexpression directly suppressed early growth response factor 1 (Egr1), resulting in a downregulated TGF-β1/Smad pathway in hepatocellular carcinoma cells [96]. The missense mutation p.H288Y of Krüppel-like Factor 2 (KLF2) in pre-clinical PAH, idiopathic PAH, and heritable PAH were indicated to reduce the expressions of MicroRNA-181a-5p (miR-181a-5p) and MicroRNA-324-5p (miR-324-5p), the exosomal miRNAs induced by KLF2. Moreover, miR-181a-5p and miR-324-5p could reduce proliferative and angiogenic responses in patient-derived cells and attenuates disease progression in PAH mice, showing the potential therapeutic role of KLF2-regulated exosomal miRNAs in PAH [38].

##### miR-455-3p-1

Enforced expression of miR-455-3p partially suppressed epithelial-to-mesenchymal transition induced by TGF-β both in breast cancer cells and tumor xenografts by directly inhibiting key components of TGF-β signaling [40]. This observation suggested that miR-455-3p was one of the regulators of the TGF-β signaling pathway. In a previous report, the differential expression of genes between the tissue of normal and PAH patients was analyzed by a microarray assay. The results suggested that microRNA-455-3p-1 (miR-455-3p-1) was downregulated in PAH patients. MiR-455-3p-1 upregulation was associated with reduced mRNA and protein levels of core RAS/ERK signal genes, suggesting that miR-455-3p-1 might involve the inhibition of the RAS/ERK pathway. In addition, upregulation of miR-455-3p-1 could inhibit the proliferation of PASMCs and alleviate PAH in vivo [97].

##### miR-223-3p

The association between microRNA-223-3p (miR-223-3p) and TGF-β signaling pathway had been studied. Bioinformatics predicted that miR-223-3p bound directly to the IncRNA ADAMTS9-AS2 and TGFBR3. ADAMTS9-AS2 transfection increased TGFBR3 mRNA and protein expressions in lung cancer cells, but miR-223-3p transfection significantly suppressed TGFBR3 expression. MiR-223-3p promotes proliferation, migration, and invasion of lung cancer cells by targeting TGFBR3 [41]. A recent report showed that the PAH pathological features in rats was alleviated as miR-223-3p overexpression and integrin subunit beta 3 (ITGB3) knockdown. It might reveal the role of miR-223-3p in PAH via ITGB3 in the extracellular matrix (ECM) pathway [98]. The direct evidence is required to demonstrate whether miR-223-3p can alleviate PAH via TGF-β signaling pathway.

#### miRNA related to BMPR2 signaling pathway in PAH

##### miR-29

The elevation of the miR-29 family was found to be associated with energy metabolism among HPAH patients. Metabolite 16α-hydroxyestrone (16αOHE), one of the estrogens, can promote the development of HPAH through upregulation of miR-29; the improvement of HPAH in Bmpr2 mice after treated with anti-miR-29, revealing the potential of this miRNA as a therapeutic target of HPAH [39]. Since miR-29 was found to associate with both pulmonary fibrosis and HPAH, it may also be a potential therapeutic target for PAH.

##### miR-204

The inflammation and increased proliferation and survival of PASMCs have provided more understanding of PAH pathogenesis [43]. In addition, the increased activation of BMPR2-mediated signal transducer and activator of transcription 3 (STAT3) has been found in PASMC isolated from PAH patients [43]. Another report revealed that the disturbance of miR-204 expression played a key in the activation of STAT3/NFAT signaling, a signaling axis involved with PAH [99].

##### miR-130/301 family

Heritable forms of PH have been defined as WHO group 1, consisting of individuals suffering from PAH, stemming either from idiopathic and hereditary forms or secondarily from co-morbidities such as congenital heart disease, autoimmune disease, drug and toxin exposure, or infections [100]. Loss-of-function mutations in the BMPR2 gene account for over 80% of hereditary PAH cases and approximately 20% of idiopathic PAH cases [101].

A previous study reveals the role of miRNAs in the integrated control of PH pathogenesis, such as the proliferative and vasoconstrictive actions of the miR-130/301 family in PH [44, 45]. Besides, miR-130/301 modulated apelin-miR-424/503-FGF2 signaling in endothelial cells while modulated STAT3-miR-204 signaling in smooth muscle cells to promote PH-associated phenotypes.

Pathogenic gene mutations from the TGF-β/BMP signaling pathways have been identified and providing compelling evidence for a central role of dysregulated BMP signaling in PAH pathogenesis [102]. However, less is known about effectors and mechanisms that might regulate vascular stiffness by modulating ECM production/composition, and the molecular mechanisms controlling these processes are still under the research [46].

##### miR-424(322)

A previous study showed that miR-424(322) secreted by pulmonary arterial endothelial cells (PAECs) could target SMURF1 and sustain BMPR2 signaling [47]. Besides, an association between circulating miR-424(322) levels and the stage of right ventricle hypertrophy, as well as an inverse correlation between miR-424(322) and SMURF1 levels in the hypertrophied right ventricle, was found through the monocrotaline rat model of PH [47]. Therefore, due to its direct effect on heart function and correlation to sustaining BMPR2 signaling, miR-424(322) might has diagnostic value in PAH patients.

##### miR-17/92 cluster

The expression of BMPR2 could be modulated by the miRNA cluster 17/92 (miR-17/92), and persistent activation of STAT3 could induce miR-17/92 expression and leads to repressed protein expression of BMPR2. Therefore, it might involve the pathogenesis of PH through BMPR2 signaling [42].

##### miR-20a

miR-20a has been reported being targeted explicitly in an in vivo model for PH, and antagomiR-20a could restore functional levels of BMPR2 in pulmonary arteries. It might also be developed to prevent the development of vascular remodeling [49].

### Comparison of the different PAH model in previous studies

Some previous studies have used at least two PAH models to confirm their findings, such as PASMCs and animal models. However, the consistency of miRNA expression might be different due to various validation systems in the same study. Therefore, to validate the existence of inconsistency, we compared these PAH models of each miRNA used in the same study as listed in Table 3. The results showed high consistency of cell, animal, and human PAH models in and between those previous studies.

**Table 3:** The difference of miRNA expression among cell, animal, and human PAH models presented in previous studies

miRNA	Cell model	Animal model	Human PAH model	Consistency	Reference
miR-21	Up (PAECs) UP (Spinal fibroblasts)	–	Up UP (skin tissue)	Yes	[22–26]
miR-29a-3p	Down (hypoxia-induced pulmonary adventitial fibroblasts)	–	–	N/A	[77]
miR-145	Up (PASMCs under hypoxic conditions)	Up (hypoxia-induced PAH rats)	Up (PAH patients under hypoxic conditions)	Yes	[88]
miR-124	–	–	Up (Specimens of PAH patients)	N/A	[34]
miR-143	UP (PASMCs)	UP (PAH calf models)	UP (lung tissues of PAH patients)	Yes	[94]
miR-29	–	Up (Bmpr2 mutant mice lungs)	Up (HPAH patients, lung tissue)	Yes	[39]
miR-223-3p	Down (PASMCs of rat with hypoxia induction)	Down (PAH rats with hypoxia induction)	–	Yes	[98]
miR-204	Down (PAH-PASMCs)	Down (PAH rat model)	–	Yes	[99]
miR-130/301 family	Up (PAECs, PASMCs of PAH mice)	**Up (hypoxic PAH mouse model)**	Up (PAH patients)	Yes	[44]

Abbreviation: *Up* upregulation of miRNA, *PAECs* pulmonary artery endothelial cells, *Down* downregulation of miRNA, *CTEPH* chronic thromboembolic pulmonary hypertension, *HPAH* heritable pulmonary arterial hypertension, *ECFCs* endothelial colony-forming cells

## Conclusions and perspectives

This study reviews the miRNAs in PAH and SSc reported in the past decade which might led to exploring a scientific insight into the SSc-PAH and PAH. The increasing knowledge of miRNAs has molded our collective appreciation of the daunting complexity of miRNA-based regulation of gene expression in this disease. Emerging trends in our understanding of the role of miRNAs in the pathogenesis of PAH and SSc might lead to novel diagnostic and therapeutic strategies for the treatment of SSc-PAH or PAH. Increasing literatures focusing on discovering molecular effectors mediating SSc-PAH pathogenesis, including large numbers of miRNA molecules expressed in pulmonary vascular cell types and system-wide regulatory functions in vascular health and disease. However, due to the inherent pleiotropy, overlap, and redundancy of these molecules, it has been challenging to define their integrated effects on overall disease manifestation. This review summarizes our current understanding of the roles of TGF-b/BMPR2 signaling pathway-related miRNAs in PAH and SSc pathology, emphasizing potential biomarkers and/or therapeutic targets for the disease. In some ways, the complexity of the hierarchical motifs governing their multifunctional and interconnected activities has brought more confusion to the precise, organized structure of miRNA-based mechanisms that drive disease. To overcome those deficiencies, the next phase of research and discovery will necessitate a pipeline of systematic endeavors designed to catalog and identify the hierarchy of activity inherent in these molecular networks. If successful, that next level of insight should further invigorate interest from academia, federal, and industry partners to pursue the collaborative development of more effective miRNA-based diagnostics and therapeutics based on such systems-level understanding of this disease.

## Data Availability

The datasets used during the current study are available from the corresponding author on reasonable request.

## Abbreviations

PAH: Pulmonary arterial hypertension
CTD: Connective tissue disease
SSc: Systemic sclerosis
miRNAs: MicroRNA
IPAH: Idiopathic pulmonary arterial hypertension
SSc-PAH: SSc-associated pulmonary arterial hypertension
TGF-β: Transforming growth factor-beta
BMPR2: Bone morphogenetic protein receptor type II
mPAP: Mean pulmonary artery pressure
RV: Right ventricular
BMP: Bone morphogenetic protein
MCT: Monocrotaline
TGF-βR1: Transforming growth factor-beta receptor type 1
MCT-PH: Monocrotaline-induced pulmonary hypertension
PASMC: Pulmonary artery smooth muscle cell
BMPR1: Bone morphogenetic protein receptor type I
TNF-α: Tumor necrosis factor α
IL-6: Interleukin 6
PH: Pulmonary hypertensive
HPAH: Heritable pulmonary arterial hypertension
PDCD4: Programmed cell death 4
Bcl-xL: B cell lymphoma-extra large
DDAH1: Dimethylarginine dimethylaminohydrolases 1
AXIN2: Axis inhibition protein 2
SMAD2: Mothers against decapentaplegic homolog 2
JNK1: c-Jun N-terminal kinase 1
ETS-1: ETS proto-oncogene 1
COL1A1: Collagen Type I Alpha 1 Chain
SSc-ILD: SSc-systemic sclerosis associated interstitial lung disease
Dnmt1: DNA methyltransferase 1
SOD2: Superoxide dismutase 2
SMURF1: SMAD-specific E3 ubiquitin protein ligase 1
ABCA1: ATP-binding cassette subfamily A member 1ITGB3 integrin subunit beta 3
PTBP1: Polypyrimidine tract-binding protein
PKM2: Pyruvate Kinase M2
NFAT: Nuclear factor of activated T cells
KLF2: Krüppel-like Factor 2
ITGB3: Integrin subunit beta 3
ECM: Extracellular matrix
16αOHE: 16α-hydroxyestrone
STAT3: Signal transducer and activator of transcription 3
PAECs: Pulmonary artery endothelial cells
